# Genome sequencing and analysis of the raccoon variant rabies lyssaviruses directly from clinical samples, Connecticut, 2017–2019

**DOI:** 10.3389/fvets.2022.1001204

**Published:** 2022-09-23

**Authors:** David H. Chung, Zeinab Helal, Julia Desiato, Holly McGinnis, Maureen Sims, Amelia Hunt, Junwon Kim, Guillermo R. Risatti, Dong-Hun Lee

**Affiliations:** ^1^Department of Pathobiology and Veterinary Science, College of Agriculture, Health and Natural Resources, University of Connecticut, Storrs, CT, United States; ^2^Connecticut Veterinary Medical Diagnostic Laboratory, Department of Pathobiology and Veterinary Science, College of Agriculture, Health and Natural Resources, University of Connecticut, Storrs, CT, United States; ^3^College of Veterinary Medicine, Konkuk University, Seoul, South Korea

**Keywords:** rabies, multiplex RT-PCR, next-generation sequencing, genome assembly, phylogenetics

## Introduction

Rabies lyssavirus is a zoonotic RNA virus typically transmitted through the bite of an infected animal *via* saliva. Infections attack the central nervous system and may result in symptoms such as respiratory arrest, paralysis, encephalitis, coma, or fatality if the patient is not treated with post-exposure prophylaxis promptly after being exposed (1). The virus is responsible for annual 59,000 human deaths globally, exhibiting great diversity in viral epidemiology and reservoir species distribution (2). Approximately 5,000 animal rabies cases are reported to the Centers for Disease Control and Prevention (CDC) each year, and most of the cases in the United States (U.S.) are from wild animals, which threaten transmission to domestic animals and even to humans (3, 4).

Lyssaviruses are detected in at least 30 identified reservoir species, consisting primarily of terrestrial carnivore species, including raccoons skunks and bats, and distributed worldwide in the Americas, Europe, Asia, Africa, and Australia (5, 6). In North America, the raccoon variant rabies lyssavirus (RRV) is of concern, given its relatively rapid spread in wildlife and its potential public health impact (7). In the U.S., the RRV's endemic geographic range has expanded in recent decades throughout the country's eastern seaboard (8). RRV was first reported in Connecticut in March 1991, when a rabid raccoon was found in Ridgefield, which borders New York State (9); since that, the RRV has spread throughout the state. In 2018 Connecticut reported 15.4% increase in the number of raccoon rabies cases detected compared with the number seen in 2017 (5).

Next-generation sequencing (NGS) approaches enable whole genome sequencing (WGS) of pathogens directly from clinical samples without isolation. Despite the potential and recent successes of whole genome metagenomics, target enrichment for specific viruses is often required to generate complete viral genome coverage while sequencing directly from clinical samples. The tiling amplicon panel schemes have been used to generate complete coverage of viral genomes (10, 11). For rabies lyssavirus, reverse transcription polymerase chain reaction (RT-PCR) based virus-specific genome enrichment methods have been used for complete genome sequencing (12, 13). Here, we optimized a multiplex tiling RT-PCR protocol with selective primer sets covering the entire coding region (CDS) of RRV in three reactions and successfully sequenced and analyzed 42 RRV-positive animal brain samples identified from Connecticut during 2017–2019.

## Materials and methods

### Rabies lyssavirus samples

In this study, we have used brain tissue samples from rabies cases submitted between 2017 and 2019 (Supplementary Table 1) to the Connecticut Veterinary Medical Diagnostics Laboratory (CVMDL). A total of 42 rabies-positive brain tissue samples of raccoon (*n* = 28), skunk (*n* = 6), woodchuck (*n* = 2), feline (*n* = 2), bobcat (*n* = 1), fox (*n* = 1), cow (*n* = 1), and deer (*n* = 1) confirmed rabies positive at CVMDL by direct fluorescent antibody test (14) and quantitative reverse transcription PCR (RT-qPCR) assay (15) were used for sequencing purposes.

### Comparative *in-silico* selection of rabies primers

The selected primer sets targeting rabies lyssavirus were designed based on previous studies (13, 16). We retrieved 1,798 reference rabies viral genome sequences from North America encompassing the period 2009–2019 available on Virus Pathogen Database and Analysis Resource (www.viprbrc.org). The viral genome dataset was subsampled to 307 sequences using nucleotide sequence identity of 98.5% on the CD-HIT server (17). Sequences were aligned using Multiple Alignment with Fast Fourier Transformation (MAFFT) (18). RT-PCR primers for rabies lyssavirus reported previously were tested using *in-silico* PCR analysis with FastPCR program v6.6 (19) to evaluate them for mismatches and annealing temperature (Ta). Primers lacking nucleotide base-pair mismatch to reference rabies viral sequences were selected. The annealing temperatures of the primers predicted by the *in-silico* PCR setting ranged from 56 to 67°C.

In this study, a total of six primer pairs ranging 1,675–2,465 bp in amplicon sizes were used to cover the complete genome of RRVs. The forward-reverse primer pairs were then re-organized into 3 distinct sets for multiplex RT-PCR reactions, including Set 1; RVfor3-PR2a pair and LF8a-RVrev2 pair, Set 2; PF2a-RRVArev pair and LF3-RRVBrev pair, and Set 3; RRVBfor-LR3 pair and RRVCfor-LR8 pair (Supplementary Figure S1A; Supplementary Table S2). We assigned alternate target genome regions so that neighboring amplicons do not overlap within the same pool.

### Viral RNA extraction and multiplex tiling RT-PCR

The viral RNA was extracted from samples using the TRIzol reagent (ThermoFisher Scientific, USA) according to manufacturer's instructions. One Taq one-step RT-PCR kit (New England BioLabs, USA, Cat# E5315S) was used to amplify genomic RNA. The RT-PCR reaction included 5 μl (500–1,000 ng) RNA template of RRV-positive samples, 10 μM of each primer (1 μl forward and 1 μl reverse primer), 12.5 μl reaction mix, 1 μl enzyme mix and 4.5 μl RNase free water to obtain a total volume of 25 μl. In this protocol, all RRV genome segments were amplified simultaneously. Amplicon panel length therefore ranges 1,676–2,485 bp together covering the complete RRV genome. Cycling conditions for the respective rabies RT-PCR were conducted as described: an initial primary reverse transcription step of 30 min at 48°C, then denaturation at 94°C for 1 min, followed by 35 cycles of 94°C for 15 s, 56°C for 30 s and 68°C for 2 min, and to conclude a final elongation step at 68°C for 5 min. PCR products were checked by agarose gel electrophoresis and purified using NucleoSpin gel and PCR clean-up kit (Macherey-Nagel, PA, USA) according to the manufacturer's instructions. After purification, DNA concentration was measured using the Qubit dsDNA HS Assay Kit and the Qubit 4 fluorometer (Thermoscientific, U.S.). DNA products (Multiplex PCR sets 1-3) were normalized and pooled together.

### Genome sequencing

The Nextera DNA Flex Sample Preparation Kit (Illumina, U.S.) was used according to the manufacturer's instructions to generate multiplexed paired-end sequencing libraries of pooled PCR products of 42 samples. The dsDNA was fragmented and tagged with adapters by Nextera transposase. The fragmented PCR amplicons with added adaptor sequences enabled a 5-cycle PCR amplification to append additional unique dual index (i7 and i5) sequences at the end of each fragmented DNA for cluster formation. PCR fragments were purified on Sample Purification Beads included in the library prep kit. Fragments were analyzed on a High Sensitivity DNA Chip on the Bioanalyzer (Agilent Technologies, U.S.) before being loaded on the sequencing flow cell. Briefly, the libraries were adjusted to 1 nM concentration and equal volumes of 5 μl of each library were pooled. The pool was denatured with NaOH (0.2 N final concentration) and further diluted to 100 pM. Control library (5% PhiX library, Illumina, U.S.) was added to the pool. The library pool was loaded in the flow cell of the 151 cycle iSeq100 i1 Reagent Kit (Illumina, U.S.). The barcoded multiplexed library sequencing (2 × 151 bp) was performed on an Illumina iSeq100 platform (Illumina, U.S.).

### Genome assembly

Reference guided genome assembly was performed using the Galaxy instance (20). We created an automated genome assembly workflow that integrated software tools for quality trimming, reference mapping, and consensus calling (Supplementary Figure S1B). Residual Nextera adapters and bases with low quality scores were removed from fastq files using Trimmomatic version 0.38.0 (21) with conservative parameters, which included removing bases from each read with a quality score < %Q30 and required a minimum read length of 100 bases each. Trimmed reads were mapped to a known reference rabies genome sequence using the Bowtie2 assembler version 2.3.4.3 (http://bowtie-bio.sourceforge.net/bowtie2). The consensus genome was called using iVar consensus version 1.2.3 (22). The complete coding genome sequences of the RRVs sequenced in this study have been deposited in the GenBank under Accession Numbers ON986428-30, ON986432-41, ON986443-55, ON986457, ON986467-69, ON986471-72, ON986474, ON986476-77, and ON986479-80.

### Phylogenetic analysis

Using the Virus Pathogen Resource Database (VIPR), 916 RRVs full genome sequences were collected in North America between 2000 and 2020. A sequence from the recent RRVs collection from Connecticut was then run through the nucleotide BLAST on NCBI (https://blast.ncbi.nlm.nih.gov/Blast.cgi) and the 100 most genetically similar sequences of RRVs from surrounding states were combined with the 916 North American RRVs sequences and the newly sequenced samples from Connecticut to compare to each other. ElimDupes software (https://www.hiv.lanl.gov/content/sequence/elimdupesv2/elimdupes.html) was used to eliminate any sequences that had 99.5% similar identity level to prune down to 203 sequences. Sequences were renamed using Microsoft Excel and BioEdit software (https://projects.ncsu.edu/cbirna/links.html) to format all names consistently. A maximum-likelihood (ML) phylogeny was created *via* Randomized Axelerated Maximum Likelihood (RAxML) with rapid bootstrap iterations of 1,000 (23).

## Descriptive results

### Dataset overview and description

The rabies-specific primers used in this study were selected from multiple candidates previously published. We compared the RT-PCR primers previously reported by Nadin-Davis et al. (13) and Campos et al. (16) against the RRV genome sequences collected from North America during the period 2009–2019 using *in-silico* PCR analysis and selected six pairs of forward-reverse primers from a previous paper published by Nadin-Davis et al. in 2017 (13). We successfully amplified 1,675–2,465 bp long amplicons that cover the complete coding sequences (CDS) of 42 RRVs using three distinct multiplex tiling RT-PCR reactions followed by sequencing of the amplicons using the Illumina iSeq100 NGS platform. The entire process from viral RNA isolation to genome assembly could be completed in 30 h leading to the generation of fastq files from 42 RNA samples, including 3 h of RT-PCR, 4 h of NGS library preparation, and 22 h of iSeq100 NGS run (Supplementary Table S3).

Sequencing read quality was determined by quality scores (%Q30) from the Illumina NGS run which indicates probability of incorrect base call leading to 99.9% inferred base call accuracy. A sum of 15,376,000 reads were generated with a total %Q30 rate of 89.95%, reads with identifiable barcode were 85.87%, both having typical rates for Illumina iSeq100 dataset (https://www.illumina.com/science/technology/next-generation-sequencing/plan-experiments/quality-scores.html), and each individual barcode was found to be distributed throughout the samples. The read distribution among the 42 samples exhibited a roughly even pattern, where samples with barcodes 7 and 18 had relatively lower identified read percentages (0.0945% and 0.1147%, respectively).

Genome assembly using the Galaxy workflow allowed the successful creation of 42 consensus sequences from the fastq file format. All samples had complete CDS. The number of mapped reads varied from 8,429 to 778,612 (Supplementary Tables S1, S3). Average sequence lengths were between 11,920 and 11,921 base pairs and complete CDS was obtained for each sequence. The mean depth coverage was varied across all sequences with a minimum value of 94.7 and maximum of 13,322.5.

**Figure 1:**
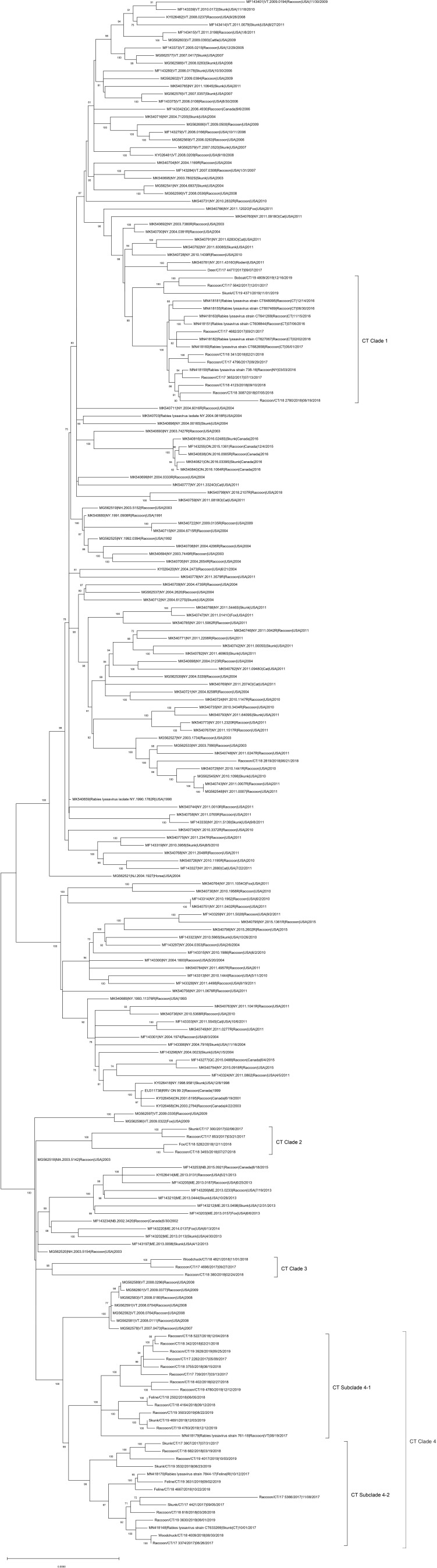
Maximum-likelihood analysis of 203 complete coding sequences of RRVs identified in North America including 42 viruses sequenced in this study. The scale bar shows the number of substitutions per site. The numerical values at each node represent 1,000 bootstrap replicate values expressed as a percentage.

The ML phylogeny indicated that RRVs in Connecticut fall into four genetically divergent subclades designated as CT Clades 1–4 (Figure 1). The viruses in CT Clade 1 detected in raccoons, skunks, bobcats, and deer clustered with viruses from cat, skunk, raccoon, and rodent from New York. The viruses in CT clades 2–4 were detected in raccoon, skunk, feline, fox, and woodchuck showed close relationship with RRV from New England regions including Vermont, Massachusetts, Maine, New Brunswick (Canada), and Rhode Island. The Deer/CT/17-4477/2017 virus did not cluster together with viruses from Connecticut, but clustered with a 2011 rodent RRV from NY (MK540781|4316O|Rodent) as an outlier.

## Discussion

Based on the 2019 rabies surveillance in the U.S., raccoons accounted for the highest percentage (32.9%) among all detected rabid wild animals (*n* = 94,770) followed by skunks (24). Consistent with previous findings (7, 24, 25), the high number of detections in raccoons and skunks in Connecticut during 2017–2019 suggests a key role in maintenance and spread of RRVs in the Northeastern U.S. These species are highly adaptive to urban environment raising the risk of rabies exposure to human and animal populations. Therefore, efficient genetic tools are in need for investigating RRV outbreaks if they should occur.

Based on our data and experience, rabies positive samples confirmed by direct fluorescent antibody test contain sufficient amount of viral RNA, mostly *Ct*-value < 30 in RT-qPCR, for multiplex tiling RT-PCR and genome sequencing. Our newly optimized multiplex tiling RT-PCR protocol was utilized with a pre-selected primer panel set that cover the complete CDS of the RRVs via three amplification reactions. While conventional NGS protocols involve high cost, labor, and complexity in the processes, our approach is both time-saving and cost-effective. The multiplex tiling RT-PCR combined with an affordable benchtop Illumina NGS system significantly enhanced experimental efficiency. The approach enables selective enrichment of viral genome from clinical samples with much less hands-on time and experimental steps.

The use of the described protocol allowed a fast and efficient genome sequencing of circulating RRV in the US northeast. The CDS-based ML phylogenetic analysis showed a high genetic diversity between RRVs detected in the State of Connecticut and those detected in neighboring states. Data generated here suggest possible viral transmissions of RRVs between Connecticut and in other States in Northeastern U.S. through movements of wild animals and independent evolution of each virus subclade in Connecticut.

In summary, we report passive surveillance WGS data for RRV detected in the state of Connecticut during 2017–2019 using our multiplex RT-PCR approach. The phylogenetic analysis combined with the newly optimized methodology provide a useful methodological tool for future RRV surveillance that includes assessments of evolution and transmission of RRV in North America.

## Data availability statement

The datasets presented in this study can be found in online repositories. The names of the repository/repositories and accession number(s) can be found in the article/Supplementary material.

## Author contributions

Conceptualization, supervision, and funding acquisition: D-HL and GR. Methodology: DC and ZH. Sample preparation: HM, MS, AH, and ZH. Data analysis: DC, JD, and JK. Data curation: AH and ZH. Writing—original draft preparation: DC. Writing—review and editing: GR, JD, HM, MS, and D-HL. All authors have read and agreed to the published version of the manuscript.

## Funding

This work was supported by the University of Connecticut's Office of the Vice President for Research (OVPR) through the Research Excellence Program.

## Conflict of interest

The authors declare that the research was conducted in the absence of any commercial or financial relationships that could be construed as a potential conflict of interest.

## Publisher's note

All claims expressed in this article are solely those of the authors and do not necessarily represent those of their affiliated organizations, or those of the publisher, the editors and the reviewers. Any product that may be evaluated in this article, or claim that may be made by its manufacturer, is not guaranteed or endorsed by the publisher.

## Supplementary material

Supplementary Figure S1 Schematic representation of the complete protocol for multiplex tiling RT-PCR and reference guided genome assembly pipeline. **(A)** Representation of the 6 primer pairs ranging 1,675–2,465 bp in amplicon sizes used to cover the complete genome of RRVs. The forward-reverse primer pairs were designed into 3 distinct sets for multiplex RT-PCR reactions (Set 1: RVfor3-PR2a pair and LF8a-RVrev2 pair, Set 2: PF2a-RRVArev pair and LF3-RRVBrev pair, and Set 3: RRVBfor-LR3 pair and RRVCfor-LR8 pair); **(B)** description of automated workflow pipeline on the Galaxy instance used for reference guided genome assembly of raccoon variant rabies samples.

Supplementary Table S1 Summary of Raccoon variant rabies virus samples and genome assembly data.

Supplementary Table S2 Multiplex PCR primer pair sets for Raccoon variant Rabies RT-PCR.

Supplementary Table S3 Summary statistics for sequencing experiment of raccoon variant rabies lyssavirus samples using Illumina iSeq100 platform.
