# Supplementary figures and images for: Genome sequencing and analysis of the raccoon variant rabies lyssaviruses directly from clinical samples, Connecticut, 2017–2019
Source: Front Vet Sci. 2022 Sep 23;9:1001204. doi: 10.3389/fvets.2022.1001204 (PMC9539882; doi:10.3389/fvets.2022.1001204)

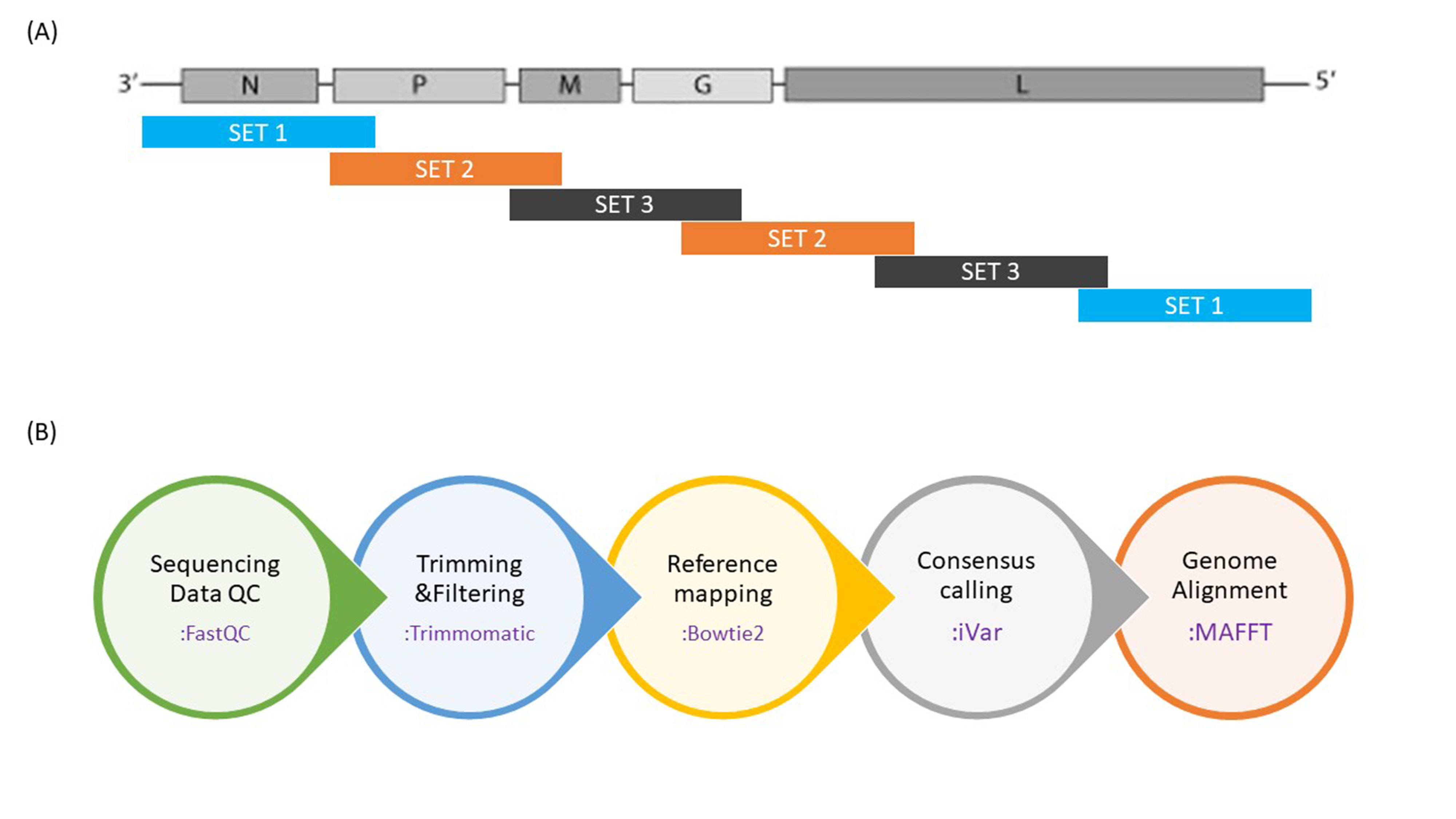

Supplement: Supplementary Figure S1 — Schematic representation of the complete protocol for multiplex tiling RT-PCR and reference guided genome assembly pipeline. (A) Representation of the 6 primer pairs ranging 1,675–2,465 bp in amplicon sizes used to cover the complete genome of RRVs. The forward-reverse primer pairs were designed into 3 distinct sets for multiplex RT-PCR reactions (Set 1: RVfor3-PR2a pair and LF8a-RVrev2 pair, Set 2: PF2a-RRVArev pair and LF3-RRVBrev pair, and Set 3: RRVBfor-LR3 pair and RRVCfor-LR8 pair); (B) description of automated workflow pipeline on the Galaxy instance used for reference guided genome assembly of raccoon variant rabies samples. [file Image_1.JPEG]
